# Retraction: Regulation of the Proteasome by AMPK in Endothelial Cells: The Role of O-GlcNAc Transferase (OGT)

**DOI:** 10.1371/journal.pone.0286133

**Published:** 2023-05-17

**Authors:** The PLOS ONE Editors

Following the publication of this article [1], concerns were raised regarding multiple figures. Specifically,

- The PA700/S10B panel in Fig 2B and OGT and PA700/S10B panels in Fig 3A appear similar despite representing different conditions.
- The PA700/S10B panels in Figs 2E and 3B appear similar.
- The PA700/S10B panels in Figs 6A, 6B, and 6C appear similar.

These issues were discussed with the first author and the corresponding author. The first author stated that some of the data underlying the results in this article are no longer available. They provided files described as the original images raw images underlying some of the panels in the above figures, however not all appeared to match the published panels.

The first author stated that the PA700/S10B panels in Figs 2B and 3A are accidental duplicates of the OGT panel in Fig 3A, and they provided a replacement panel. They also stated that the β7 panel in Fig 2B is incorrect and provided a replacement. The replacement panel provided for β7 appears to partially overlap with the β7 panel in Fig 6C despite representing different conditions.

The first author stated that the PA700/S10B panels in Figs 2E and 3B are identical and represent the same conditions. They acknowledge that the reuse of the image should have been declared in the figure legends.

The first author provided versions of the western blots in Fig 6 showing more of the gels horizontally. These versions of the images raised additional concerns about the accuracy and reliability of the data in Fig 6.

In light of the concerns affecting multiple figure panels that question the reliability of these data, the *PLOS ONE* Editors retract this article.

MHZ agreed with the retraction. JX did not agree with the retraction. SW and BV either did not respond directly or could not be reached. JX stands by the article’s findings.
